# Maximizing Post-exercise Anabolism: The Case for Relative Protein Intakes

**DOI:** 10.3389/fnut.2019.00147

**Published:** 2019-09-10

**Authors:** Daniel R. Moore

**Affiliations:** Faculty of Kinesiology and Physical Education, University of Toronto, Toronto, ON, Canada

**Keywords:** muscle protein synthesis, muscle hypertrophy, resistance training, essential amino acids, dietary protein, lean body mass, recovery

## Abstract

Maximizing the post-exercise increase in muscle protein synthesis, especially of the contractile myofibrillar protein fraction, is essential to facilitate effective muscle remodeling, and enhance hypertrophic gains with resistance training. MPS is the primary regulated variable influencing muscle net balance with dietary amino acid ingestion representing the single most important nutritional variable enhancing post-exercise rates of muscle protein synthesis. Dose-response studies in average (i.e., ~80 kg) males have reported an absolute 20 g dose of high quality, rapidly digested protein maximizes mixed, and myofibrillar protein synthetic rates. However, it is unclear if these absolute protein intakes can be viewed in a “one size fits all” solution. Re-analysis of published literature in young adults suggests a relative single meal intake of ~0.31 g/kg of rapidly digested, high quality protein (i.e., whey) should be considered as a nutritional guideline for individuals of average body composition aiming to maximize post-exercise myofibrillar protein synthesis while minimizing irreversible amino acid oxidative catabolism that occurs with excessive intakes of this macronutrient. This muscle-specific bolus intake is lower than that reported to maximize whole body anabolism (i.e., ≥0.5 g/kg). Review of the available literature suggests that potential confounders such as the co-ingestion of carbohydrate, sex, and amount of active muscle mass do not represent significant barriers to the translation of this objectively determined relative protein intake. Additional research is warranted to elucidate the effective dose for proteins with suboptimal amino acid compositions (e.g., plant-based), and/or slower digestion rates as well as whether recommendations are appreciably affected by other physiological conditions such endurance exercise, high habitual daily protein ingestion, aging, obesity, and/or periods of chronic negative energy balance.

## Introduction

Lean body tissues, including skeletal muscle, are constantly being remodeled through the continuous and simultaneous processes of protein synthesis, and protein breakdown (collectively referred to as “turnover”). This constant turnover functions to breakdown old and/or damaged proteins and synthesize new proteins to help maintain protein mass and quality. Importantly, the algebraic difference between synthesis and breakdown determines net protein balance of a given tissue (e.g., muscle) and, ultimately, whether it is gaining or losing mass. To this end, resistance exercise increases muscle protein turnover for up to 48 h in the fasted state (1). Due to the greater stimulation of muscle protein synthesis compared to breakdown, muscle net balance is improved but, in the absence of exogenous amino acids, remains in a net negative balance (1, 2). It is only until a source of exogenous amino acids that net balance becomes positive due primarily the enhancement of muscle protein synthesis (3). Ultimately, the synergistic effects of resistance exercise and amino acid ingestion provides the requisite anabolic environment to support net tissue growth (i.e., muscle hypertrophy) characteristic of resistance training.

Notwithstanding the technical and logistical challenges associated with measuring rates of muscle protein breakdown in humans (especially in the postprandial state) (4), muscle protein synthesis is generally regarded as the prime-regulated variable in healthy humans in response to exercise and/or nutrition (5, 6). For example, the characteristic increase in muscle protein breakdown that occurs after resistance exercise in the fasted state is negated by the provision of exogenous amino acids, which subsequently supports greater rates of muscle protein synthesis, and an increased (and positive) net protein balance (3). The post-exercise increase in muscle protein synthesis that occurs with the ingestion of different dietary proteins (e.g., milk vs. soy) has also been shown to qualitatively predict training-induced increases in muscle hypertrophy and lean mass gains in young individuals (7–9). Importantly, measurement of the contractile myofibrillar protein subfraction, which is preferentially enhanced by resistance exercise and protein/amino acid ingestion (10–12), enhances the predictive ability of long-term (i.e., 24–48 h) rates of synthesis for muscle hypertrophy (13). Thus, identifying nutritional factors that may augment the exercise-induced increase in myofibrillar protein synthesis during this prolonged (i.e., >24 h) recovery period would ostensibly be an effective strategy to promote muscle hypertrophy. Therefore, the present review will focus on how dietary protein ingestion enhances post-exercise rates of muscle protein synthesis with a focus on the contractile myofibrillar protein fraction as a means to enhance recovery from, and adaptation to resistance exercise. The overall aim of this review will be to objectively determine the “optimal” relative bolus protein ingestion during the post-exercise recovery period as defined by one that maximizes myofibrillar protein synthesis while concomitantly minimizing estimated rates of amino acid oxidation. Potential biological (e.g., sex, age, body composition, active muscle mass), and nutritional (e.g., macronutrient co-ingestion, habitual protein intake, food matrix) confounders will be discussed to explore potential translational issues with recommending a per meal relative protein intake based on a preponderance of studies in young adults utilizing an isolated protein source (i.e., whey).

## Regulation of Muscle Protein Synthesis After Exercise by Dietary Amino Acids

Since the first observations that skeletal muscle protein turnover is elevated in response to resistance exercise and that exogenous amino acids augment the increase in net protein balance of this tissue (2, 3), studies have investigated the nutritional factors that contribute to the optimal enhancement of post-exercise anabolism. This line of research has revealed that the most critical factor to enhance post-exercise muscle protein synthesis is the provision of dietary amino acids with the essential amino acids (EAA) primarily driving the response (14–17). A series of seminal studies from the Wolfe laboratory were the first to suggest a potential amino acid dose-response existed during recovery from resistance exercise in humans (15, 17, 18). These parallel studies demonstrated that lower EAA intakes (6–12 g) were associated with an apparent graded increase in muscle net balance (17, 18). When amino acid intakes were greater (i.e., 15 vs. 40 g EAA) there was a similar increase in post-exercise anabolism (15), suggestive of a potential ceiling effect. These seminal studies performed with crystalline amino acids provided the framework for future research into the nutritional regulation of post-exercise muscle protein synthesis. Importantly, as dietary amino acids are generally consumed as complete proteins, the next wave of muscle protein metabolism research investigated the ability of dietary protein to enhance post-exercise muscle remodeling.

## Absolute Protein Intake to Maximize Post-exercise MPS

**Figure 1:**
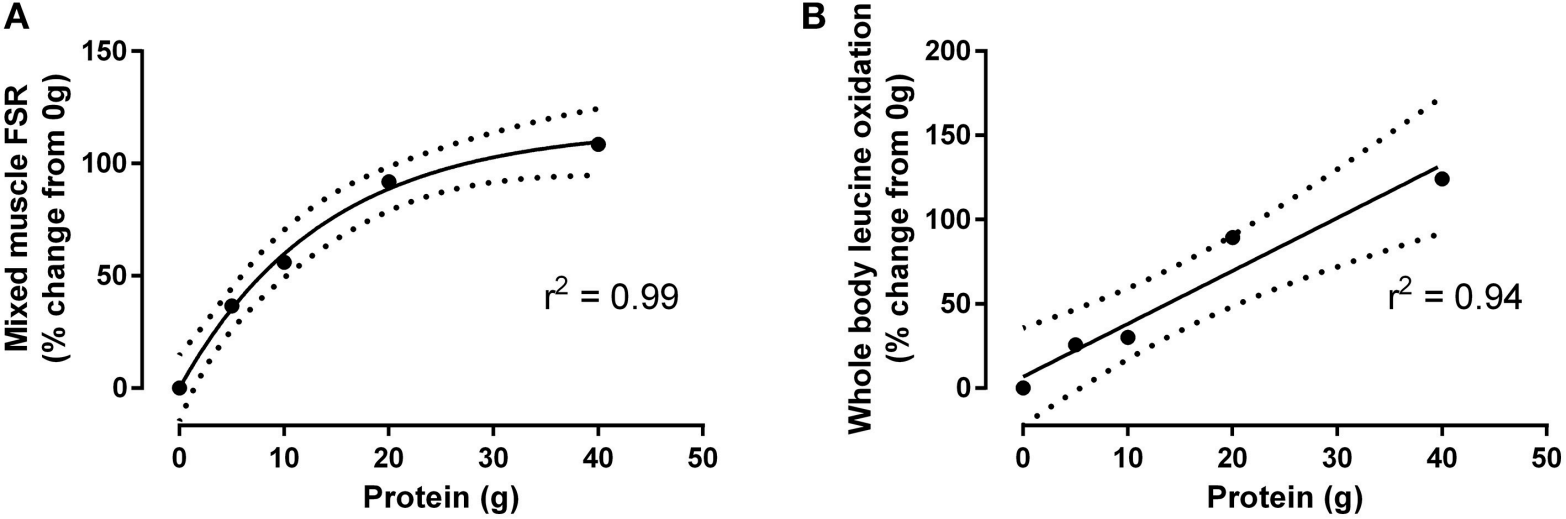
Percent-change from fasted (i.e., 0 g protein ingestion) of mixed muscle protein synthesis **(A)** and whole body leucine oxidation **(B)** after resistance exercise in response to graded intakes of egg white protein, as adapted from Moore et al. (19). Data conform to one phase-exponential decay and linear correlation, respectively (Graphpad Prism V.6). Hashed line represents 95% CI.

The first study to address the post-exercise ingested protein dose-response required healthy young resistance trained subjects with an average body mass of ~86 kg to perform a bout of heavy bilateral leg-based resistance exercise (i.e., leg press, knee extension, leg curl) before ingesting a variable amount of egg protein to enhance mixed muscle protein synthesis (19). Consistent with earlier results using crystalline amino acids (17, 18, 20), it was observed that even small amounts of protein (i.e., 5 and 10 g) were sufficient to enhance post-exercise mixed muscle protein synthesis (19). Importantly, mixed muscle protein synthesis was further enhanced by 20 g of protein but revealed an apparent plateau as a doubling of ingested protein to 40 g had no additive effect on the post-exercise protein synthetic response. These data ultimately conformed to a one-phase exponential decay relationship (Figure 1) that is characteristic of many allosterically regulated enzymes of the body, such as those within the mTOR pathway that control mRNA translation and muscle protein synthesis (21, 22), and is consistent with a ingested protein dose-response curve. It was subsequently demonstrated that the myofibrillar protein fraction displays a similar ingested protein dose-response relationship with 20 g of whey protein eliciting a maximal synthetic response (23). A unique feature of the study by Witard et al. (23) was that the post-exercise whey protein dose-response occurred ~4 h after participants consumed a high protein (~30% energy) breakfast, highlighting that the pre-exercise nutritional state (i.e., fasted vs. fed) does not appear to have a substantial impact on the post-exercise protein requirement to maximize muscle protein synthesis. This may be particular relevant for many athletes who have reported to consume ~5 daily meals and would therefore be in a postprandial state for the majority of their waking hours (24). Therefore, similar to rested skeletal muscle (23), 20 g of high quality dietary protein appears to be sufficient to support maximal post-exercise rates of muscle protein synthesis in average weight (i.e., 80–85 kg) young adult males.

In contrast to the plateau observed with muscle protein synthesis, whole body leucine oxidation (a surrogate measure of protein oxidation) increases in a linear fashion with graded protein intakes (Figure 1) (19). This linear relationship may be related to the combination of a relatively low K_m_ for the rate-controlling enzyme for leucine oxidation (i.e., branched-chain ketoacid dehydrogenase) (25), and a greater overall substrate supply (i.e., leucine, valine, and isoleucine) with higher protein intakes. Importantly, this increase in leucine oxidation in conjunction with a concomitant increase in urea synthesis (23) highlights that dietary amino acids provided at levels that are in excess of their ability to be incorporated into new (muscle) proteins results in their deamination and, in the case of the branched chain amino acids, irreversible oxidation (Figure 1). This pattern of dietary amino acid oxidation is arguably an inefficient use of ingested protein if the specific goal is to maximize post-exercise muscle protein synthesis and anabolism. In fact, the marked increase in whole body leucine oxidation concomitant with a sustained elevation in blood amino acid concentration (19) is consistent with a metabolic pattern that has been suggested to be characteristic of an upper limit of intake for this macronutrient (26). Therefore, given the ability to induce a plateau in muscle protein synthesis yet minimize amino acid oxidation and urea synthesis (19, 23), 20 g of high quality protein (e.g., egg or whey protein) arguably represents an “optimal” or absolute protein intake to efficiently enhance muscle remodeling after resistance exercise in young adults.

## Relative Protein Intake to Maximize Myofibrillar Protein Synthesis

Based on previous studies that provided absolute protein intakes, the ingestion of 20 g of protein that was shown to maximize both mixed muscle and myofibrillar protein synthesis yet minimize whole body leucine oxidation, and ureagenesis in ~85 kg males translates into a relative protein intake of ~0.24 g protein/kg body weight. However, the ability to extrapolate these relative intakes into an “optimal” one-size-fits-all recommendation is arguably limited by the small sample size (i.e. *n* = 54) of “average” body mass individuals. In addition, the qualitative (albeit not statistically significant) ~10% increase in muscle protein synthetic rates between 20 and 40 g of protein could be interpreted as reflecting the “true” maximal intake as being within these two doses. Therefore, logical questions such as “would intakes greater than 20 g of protein further enhance muscle protein synthesis?” and “would 20 g of protein be the target intake for both 55 and 120 kg athletes?” naturally flow from these acute, absolute protein intake studies. In addition, recommendation of absolute meal protein intakes is at odds with daily recommendations for this macronutrient, which are almost universally prescribed relative to body mass.

To address these types of generalizability concerns, an unsystematic review was performed in Pubmed from its inception to July 1, 2019 consisting of keywords related to this review topic such as “whey,” “myofibrillar protein synthesis,” and “exercise.” As maximizing post-exercise myofibrillar protein synthesis would be essential for those interested in enhancing muscle growth and potentially strength with training, studies investigating the synthesis of this muscle fraction were selected to increase homogeneity as well as reflect the greater contractile and nutrient sensitivity of this protein fraction (12). Moreover, studies that utilized a bolus protein feeding of whey protein after exercise and measured the synthesis of the myofibrillar protein fraction by traditional primed-constant stable isotope infusion during the subsequent 3–5 h postprandial period were included. Given that the preponderance of studies fitting these criteria have been performed in young adults, only this age group (i.e., <35 y) was included in the final dose-response analysis to minimize any confounding effects of age (see below for additional discussion). Finally, given the variability in fractional synthetic rates across different stable isotopes and precursor pools (27), post-exercise myofibrillar protein synthetic rates were expressed as a change from reported (when available) or estimated basal rates to better compare across studies. Details of the studies utilized for the subsequent analysis are presented in Table 1. Only articles in English were assessed with reference lists cross-checked for any additional relevant articles.

**Table 1:** Overview of studies investigating the post-exercise stimulation of myofibrillar protein synthesis with bolus whey protein ingestion.

	**Participants**	**Body mass (kg)**	**Absolute protein intake (g)**	**Relative protein intake (g/kg)**	**Exercise modality**	**Active muscle (kg)^a^**	**Post-exercise MPS^b^**	**MPS increase^c^ (%)**
Areta et al. (28)	*n* = 8 M	81 ± 11	20	~0.25	Bilateral KE	~7.2	1–4 h	~147
Areta et al. (28)	*n* = 8 M	84 ± 11	40	~0.48	Bilateral KE	~7.4	1–4 h	~134
Burd et al. (29)	*n* = 8 M	84 ± 9	20	~0.24	Unilateral KE	~3.8	0–5 h	~166
^*^Churchward-Venne et al. (30)	*n* = 8 M	77 ± 11	25	~0.32	Unilateral KE	~3.4	0–5 h	~171
^†^MacNaughton et al. (31)	*n* = 15 M	77 ± 5	20	~0.26	Bilateral CP, LPD, LP, KE, LC	~28.1	0–5 h	~47
^†^MacNaughton et al. (31)	*n* = 15 M	77 ± 5	40	~0.52	Bilateral CP, LPD, LP, KE, LC	~28.1	0–5 h	~84
^†^MacNaughton et al. (31)	*n* = 15 M	98 ± 8	20	~0.20	Bilateral CP, LPD, LP, KE, LC	~37.4	0–5 h	~58
^†^MacNaughton et al. (31)	*n* = 15 M	98 ± 8	40	~0.41	Bilateral CP, LPD, LP, KE, LC	~37.4	0–5 h	~83
McGlory et al. (32)	*n* = 10, M	80 ± 8	30	~0.37	Unilateral LP, KE	~10.8	0–3 h	~221
McKendry et al. (33)	*n* = 8, M	83 ± 11	25	~0.30	Bilateral LP, KE	~22.3	0–4 h	~139
Moore et al. (12)	*n* = 7 M	85 ± 12	25	~0.29	Unilateral KE, LP	~11.4	0–5 h	~180
Reidy et al. (34)	*n* = 8, M	76	17.3	~0.23	Bilateral KE	~6.7	3–5 h	~166
^‡^Reitelseder et al. (35)	*n* = 9 M	79 ± 9	17.5	~0.22	Unilateral KE	~3.5	1–6 h	~103
^‡^Reitelseder et al. (35)	*n* = 8 M	74 ± 6	0	0	Unilateral KE	~3.3	1–6 h	~81
^*^West et al. (36)	*n* = 8 M	84 ± 12	25	~0.30	Unilateral BC	~2.0	0–3 h	~150
^*^West et al. (36)	*n* = 8 M	84 ± 12	25	~0.30	Unilateral BC, Bilateral LP, KE, LC	~24.7	0–3 h	~129
West et al. (37)	*n* = 8 M	80 ± 10	25	~0.31	Bilateral KE	~7.1	1–5 h	~150
West et al. (38)	*n* = 8 M	77 ± 11	25	~0.32	Bilateral LP, KE, LC	~20.8	1–5 h	~160
West et al. (38)	*n* = 8 F	67 ± 6	25	~0.37	Bilateral LP, KE, LC	~19.5	1–5 h	~124
Witard et al. (23)	*n* = 12 M	83 ± 15	0	0	Unilateral KE	~3.7	0–4 h	~59
Witard et al. (23)	*n* = 12 M	84 ± 6	10	~0.12	Unilateral KE	~3.7	0–4 h	~84
Witard et al. (23)	*n* = 12 M	83 ± 7	20	~0.24	Unilateral KE	~3.7	0–4 h	~119
Witard et al. (23)	*n* = 12 M	79 ± 10	40	~0.51	Unilateral KE	~3.5	0–4 h	~141

MPS, myofibrillar protein synthesis; M, males; F, females; KE, knee extension; LP, leg press; BC, biceps curl; LC, leg curl; LPB, latissimus pull down; VL, vastus lateralis; BB, biceps brachii.

* Control MPS estimated from Moore et al. (39), which utilized identical ring-[^13^C_6_]phenylalanine tracer methodology.

† *Control MPS rested 0 g from Witard et al. (23)*.

‡ *Control MPS estimated as median value from Smith et al. (27) for L-[^13^C]leucine infusion with [^13^C]ketoisocaproate acid enrichment as the precursor*.

a *Active muscle mass estimated by first assuming total leg skeletal muscle mass represents ~29 and ~27% of total body mass for females and males, respectively, and total arm skeletal muscle mass represents ~9.5% of total body mass for males (40). These values were then divided in half to obtain the estimated single arm and single leg muscle mass and multiplied by 0.5 for BC exercise (i.e., ~50% of total arm muscle activated during arm flexion), 0.33 for KE exercise (i.e., ~33% of total leg muscle mass activated during knee extension), and 1.0 for LP exercise (i.e., ~100% of total leg muscle mass activated during leg press). Total active muscle mass was the sum of the estimated active muscle mass for each arm and/or leg*.

b *Represents duration over which MPS was measured after exercise*.

c *MPS increase above control MPS*.

**Figure 2:**
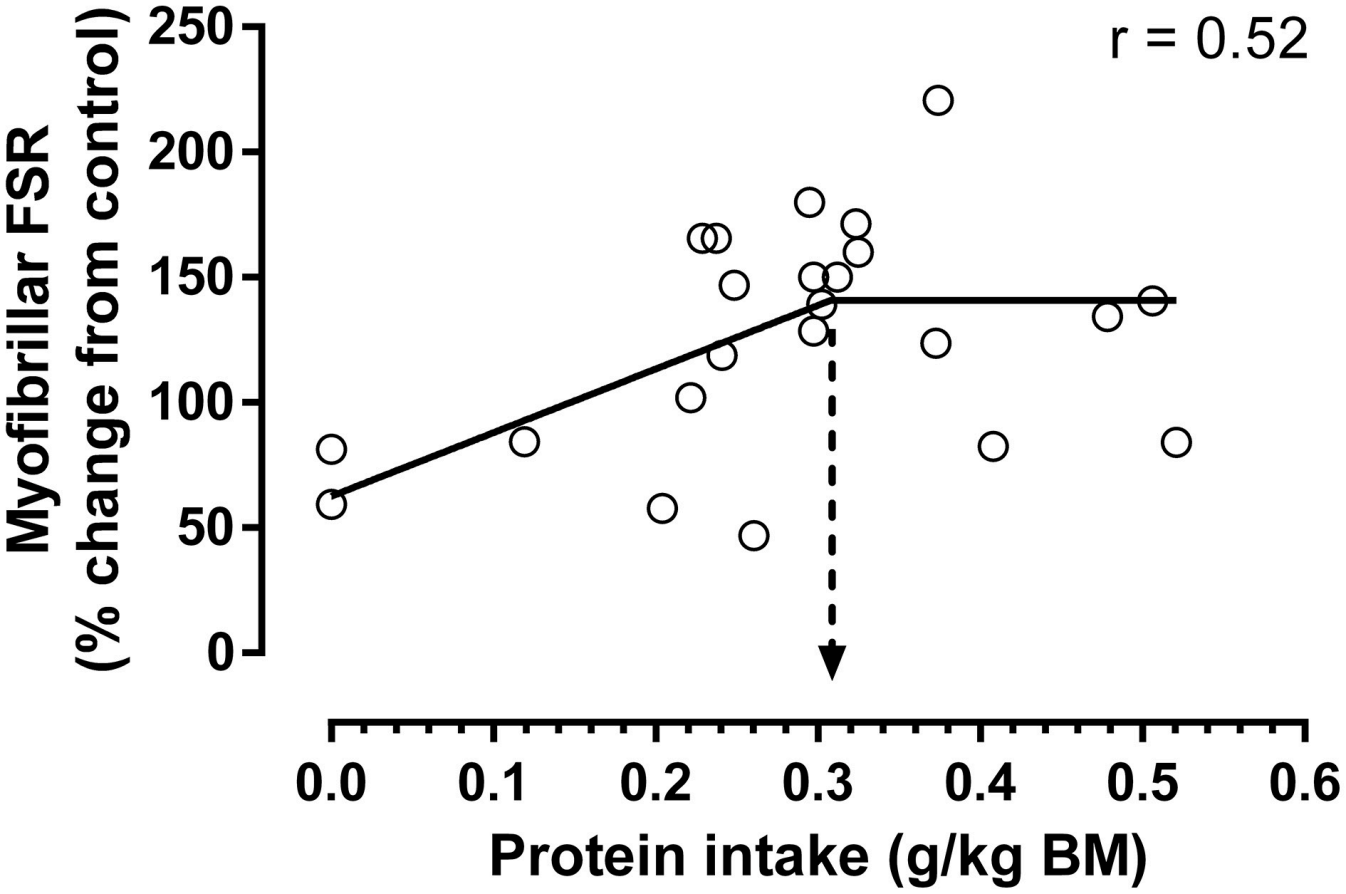
Increase in post-exercise myofibrillar protein synthesis above control relative to ingested protein normalized to total body weight (for study details, see Table 1). Bi-phase linear regression was performed with the slope of the second line segment constrained to zero and the average protein intake to maximize myofibrillar protein synthesis determined by breakpoint analysis (indicated by hashed arrow; 0.31 ± 0.08 g protein/kg body weight; mean ± SE; *N* = 23 protein intakes; analysis performed by Graphpad Prism 6.0). Applying a typical ~25% variance when analyzing individual myofibrillar protein synthetic rates (39) as compared to a collapsed mean study response, a safe intake could represent ~0.38 g/kg. There was a strong trend for a bi-phasic linear regression model to explain a greater proportion of variance vs. a simple linear regression model (*r*^2^ = 0.27 vs. 0.129, respectively; *P* = 0.056), suggesting the data conformed to a saturable dose-response relationship. First line segment described by: *y* = 254x + 63. Estimated maximal increase in myofibrillar protein synthesis above control is ~142% (as determined from equation above at 0.31 g protein/kg).

By utilizing a step-wise modeling comparison similar to our previous study at rest (39), it was observed that the increase in post-exercise myofibrillar protein synthesis in young adults with protein ingestion displayed a bi-phase linear response that is consistent with the previous observation of a dose-response relationship (Figure 2). Breakpoint analysis revealed that the bi-phase linear response plateaued at ~0.31 g protein/kg body weight (i.e., estimated average requirement), which when accounting for a typical ~25% individual response variance in young adults (39) that would not be reflected in mean study responses could result in a safe intake of ~0.39 g/kg as an upper limit. This protein intake of ~0.31 g/kg is slightly higher than the relative intake calculated from the estimated plateau in protein synthesis and average group body weight previously determined in the mixed [~0.23 g/kg; (19)], and myofibrillar [~0.24 g/kg; (23)] protein fractions after the ingestion of 20 g of protein. This could explain in part the ~10% non-significant increases in protein synthesis from the 20 to 40 g doses (19, 23), which could suggest that the 20 g dose was not sufficient to maximize protein synthesis in all subjects whereas 40 g was clearly surfeit. In fact, the apparent lack of a true plateau in previous dose-response studies had led some to suggest that the protein intake to maximize muscle protein synthesis were within this range (i.e., >20 g) and that the upper level (i.e., 40 g) was necessary to obtain a maximal anabolic response (41). However, in contrast to the suggestion that 0.4–0.5 g protein/kg lean body mass (~0.34–0.43 g protein/kg body mass, assuming an average 15% body fat) should be ingested both before and after exercise (41), the data presented herein would suggest that only a moderately higher level of protein [i.e., ~0.31 vs. ~0.24 g/kg; (23)] should be ingested to reach a plateau in post-exercise myofibrillar protein synthesis.

## Would Sex Affect the Relative Protein Requirement?

Currently, research that evaluates the nutritional factors that enhance muscle protein synthesis after resistance exercise is primarily performed in young males with that of female athletes being unfortunately under-represented. For example, the only studies to evaluate the ingested protein dose-response, either at rest or after resistance exercise, have performed these investigations in males only (19, 23, 39, 42). The reason(s) for the unfortunate disparity in sex-based research is unclear but may include, in part, the potential influence of menstrual phase on protein kinetics, which has been reported to alter the basic requirements for some EAA (e.g., lysine) at rest (43) as well as influence whole body protein metabolism during endurance exercise (44). However, the stimulation of myofibrillar protein synthesis after resistance exercise is uninfluenced by the menstrual phase (45). Moreover, both the rested (46, 47), and the exercise-induced stimulation (48) of muscle protein synthesis are similar between young men and women in the fasted state, suggesting sex *per se* has little influence on the regulation of muscle protein remodeling in the absence of any nutritional manipulation.

With respect to the nutrient sensitivity of muscle protein synthesis, seminal work that investigated the nutritional factors that enhance post-exercise muscle anabolism reported no differences between males and females in their mixed study populations (15–18); this could suggest there are no overt differences in post-exercise nutrient sensitivity of muscle protein metabolism between sexes. It has also been demonstrated that the stimulation of myofibrillar protein synthesis with resistance exercise and a 25-g bolus of dietary protein ingestion is similar between young men and women (38). This study (38) provided an absolute amount of protein (25 g) to all participants that would likely translate into a saturating dose for both the men (~0.32 g/kg) and, especially, women (~37 g/kg), which makes it difficult to determine if potential sex differences exist at lower protein intakes. Nevertheless, the ability of whey protein to enhance post-exercise rates of myofibrillar protein synthesis during energy restriction is essentially identical between females and males when normalized to fat free mass (FFM) over a range of intakes (i.e., 0–0.8 g/kg FFM) (49). Therefore, despite a relative dearth of research studying the nutritional requirements of females after resistance exercise, it is difficult to envision, based on the current literature, a scenario in which acute protein requirements would be markedly disparate between the sexes.

## Carbohydrate Co-ingestion

Carbohydrate ingestion during the recovery from resistance exercise is important for glycogen resynthesis (50, 51) and can contribute to the daily positive energy balance that is a general requisite to support muscle mass growth with training. Aside from providing additional energy during post-exercise recovery, it was first demonstrated that the co-ingestion of carbohydrate with crystalline amino acids improved post-exercise muscle net balance to a greater degree than amino acids alone (18). Subsequent studies revealed that this greater net anabolism was due primarily to an insulin-induced suppression of muscle protein breakdown rather than an augmentation of muscle protein synthesis (52, 53). In fact, as little as ~30 g of carbohydrate (and the associated insulin response) is sufficient to suppress post-exercise muscle protein catabolism (52). Provided dietary protein is provided at a level that would optimize muscle protein synthesis (i.e., ≥20 g), carbohydrate co-ingestion from 30–270 g has no additive effect on post-exercise muscle protein synthetic rates (52, 54, 55). Therefore, although it is unclear if carbohydrate co-ingestion may improve the synthetic effect of smaller (i.e., <20 g or <0.31 g/kg) amounts of dietary protein, it is clear that optimal protein ingestion is of paramount importance to maximize muscle protein synthesis after resistance exercise with mixed protein-carbohydrate beverages.

## Does the Amount of Active Muscle Mass Influence Post-exercise Protein Requirements?

It is customary for individuals engaged in resistance training for the goal of gaining muscle mass to perform whole body resistance exercise, which is in contrast to many acute studies aimed at understanding the local (i.e., muscle-specific) nutrient requirements to maximize muscle protein synthesis. This led MacNaughton et al. (31) to design an elegant study whereby groups of participants with markedly different body compositions were provided with moderate (20 g) and higher (40 g) doses of protein after a strenuous bout of whole body resistance exercise. The authors hypothesized that total lean body mass (LBM), and thus active lean (i.e., muscle) mass, would modify the acute requirement for dietary protein to maximize muscle protein synthesis during recovery. In contrast to their hypothesis and arguably the most compelling case against any impact of active muscle mass on acute protein requirements was the observation that participants with ~20 kg difference in LBM (i.e., ~59 vs. 79 kg LBM) had identical rates of myofibrillar protein synthesis after consumption of a moderate 20 g dose of whey protein. This finding is not without precedence as it has been shown previously that performing an intense bout of lower body resistance exercise (i.e., leg press, knee extension, leg curl), which would increase total body active muscle mass, does not impact blood flow during recovery to the arm nor post-exercise rates of myofibrillar protein synthesis with a moderate 25 g protein dose in the small biceps brachii (36, 56). Macnaughton et al. argued that the lower rates of myofibrillar protein synthesis in their whole body exercise protocol relative to a previous study utilizing unilateral leg resistance exercise (23) concomitant with statistically greater rates of synthesis with the larger (i.e., 40 g) dose were nevertheless indicative of greater post-exercise protein requirement with a greater active muscle mass (23). However, the study and cohort differences in myofibrillar synthesis rates are within the general inter-study variability (i.e., ±25%) for tracer-derived rates of human muscle protein synthesis (27). Arguably the most plausible reason for the greater myofibrillar synthetic rates with 40 as compare to 20 g of protein would be a greater statistical power to detect the relatively small ~20% difference between conditions, which the authors allude to in their discussion (31). For example, *post hoc* power analysis of previous absolute protein dose-response studies (19, 23) suggest that ~35 participants would be required to achieve statistical significance for the ~10% greater muscle protein synthetic rates with 40 g as compared to 20 g protein ingestion. This is markedly similar to the results in MacNaughton et al. (31) given that statistical significance between 20 and 40 g of protein was only achieved when the low and high LBM cohorts were collapsed (i.e., *n* = 30 total).

**Figure 3:**
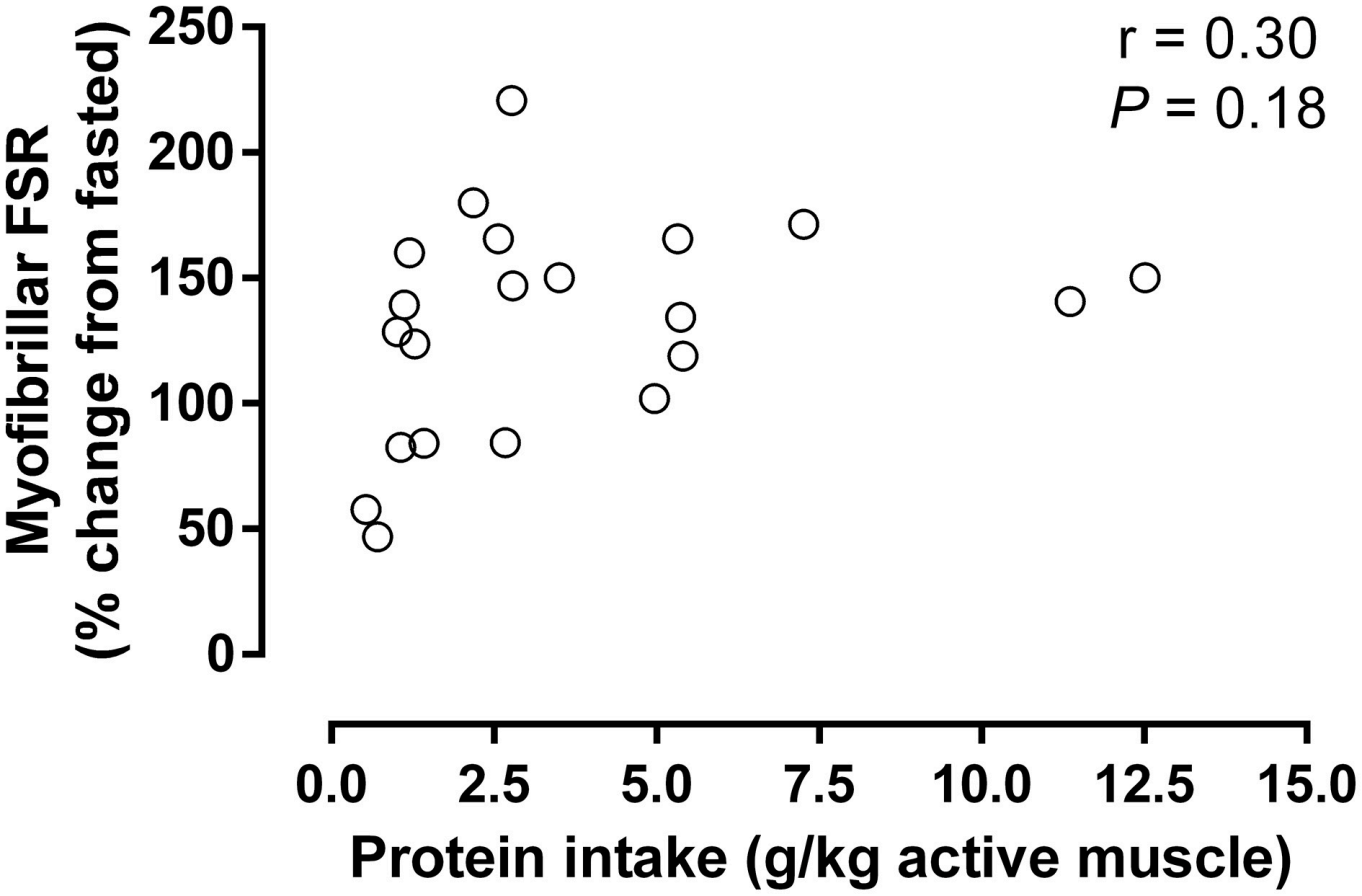
Increase in myofibrillar protein synthesis above control after resistance exercise compared to ingested protein normalized to the estimated active muscle mass (for details, see Table 1). Data were analyzed using a linear correlation (Graphpad Prism V6). Non-significant slope defined by: *y* = 3.91x + 116 (*r* = 0.30; *P* = 0.18; *N* = 21 as only conditions with protein ingestion were included).

In order to more objectively estimate the impact of the amount of active muscle mass on post-exercise protein requirements, the increase in myofibrillar protein synthesis was compared to the amount of dietary protein ingested relative to the estimated active muscle mass (Table 1; Figure 3). If one were to expect the amount of active muscle mass influenced the ability of dietary protein to stimulate post-exercise muscle remodeling, then it would be likely that a greater protein intake per active muscle mass would also result in a greater increase in myofibrillar protein synthesis. Despite a greater than ~10-fold difference in relative protein intakes there was no observable relationship with the stimulation of myofibrillar protein synthesis, which suggests active muscle mass has little bearing on post-exercise protein requirement. The observation that the stimulation of muscle protein synthesis is apparently unrelated to the amount of protein ingested per unit of active muscle is not surprising given that resistance exercise is inherently anabolic and has been shown to improve intracellular amino acid recycling (1, 2). This enhanced intracellular amino acid reutilization would ultimately lessen the requirement for exogenous amino acids to support the exercise-induced stimulation of muscle protein synthesis, although protein/amino acid ingestion is still required to induce a net positive muscle protein balance. Therefore, presently available data suggest that the amount of active muscle mass has little bearing on the ability of or requirement for post-exercise protein ingestion to enhance muscle protein remodeling.

## What About Maximizing Whole Body Anabolism?

During the post-exercise recovery period muscle protein synthesis is maximized with the ingestion ~0.31 g/kg of protein whereas muscle protein breakdown has been demonstrated to be maximally suppressed with a moderate insulin response (e.g., from ~30 g of carbohydrate) (52). Collectively, this provides compelling evidence that muscle protein net balance is saturable and primarily dictated by the nutritional enhancement of rates of muscle protein synthesis, as highlighted previously (6). In contrast, it has recently been suggested that there is no practical maximal anabolic response to dietary protein at the whole body level given the hypothesized role of an inexhaustible ability to suppress protein breakdown at high protein intakes (57, 58). For example, ingesting 70 g (~0.82 g/kg) as compared to 40 g (~0.48 g/kg) of dietary protein has been shown to enhance whole body net protein balance, in the absence of any further increase in rested or post-exercise rates of mixed muscle protein synthesis, through a proportional reduction in estimates of whole body protein breakdown (59). Based on these findings as well as those from older adults (60), the authors recently collapsed their data across ages and reanalyzed using a linear model to support their suggestion of there being no practical limit (58). This appeared to confirm their previous hypothesis on this topic (57) and potentially influenced previous suggestions of a target meal protein intake for resistance-trained athletes of ~0.4–0.55 g/kg (61). However, we recently demonstrated that whole body net balance plateaus with dietary protein ingestion after resistance exercise in females (62) and variable intensity stop-and-go exercise in both sexes (63) despite a linear increase in estimates of amino acid deamination (i.e., increased urinary urea:creatinine ratio), and presumably oxidation. The apparent discrepancy may be related in part to the choice of statistical model in our research (62, 63) as compared to others (58) (i.e., biphase vs. linear regression, respectively). In potential support, extraction and reanalysis of whole body net protein balance data from just their young adults relative to body weight-normalized protein ingestion from Kim et al. (58) revealed that the data is better fit by a segmental bi-phase linear regression as compared to standard linear model (i.e., *r*^2^ = 0.62 vs. 0.53, respectively; *P* < 0.05; Graphpad Prism V6). This analysis revealed a breakpoint in whole body net balance at ~0.71 g/kg, which is slightly greater than our recent estimates of ~0.5–0.6 g/kg (62, 63), and suggests that the capacity to assimilate dietary protein at the whole body level is substantially greater than at the muscle. While it has been suggested that these amino acids sequestered at the whole body level (e.g., within splanchnic tissues and/or circulating proteins) may be made available for muscle protein synthesis during the post-absorptive period (58), this possibility has yet to be empirically demonstrated. Some may also view these relatively higher per meal protein estimates as being unrealistic, although many Western populations with a skewed daily protein distribution routinely consume on average ~0.55 g/kg in their evening meal (64). Therefore, in contrast to prior suggested meal protein intakes of up to ~0.5 g/kg that are based on the supposition of no maximal whole body anabolism (61), it is argued that a more prudent “muscle-centric” target that maximizes muscle protein synthesis yet minimizes excess amino acid oxidative losses would place a more efficient intake at no more than ~0.39 g/kg.

## Potential Caveats to Acute Relative Protein Requirements

The reanalysis of the relative protein intake to maximize post-exercise myofibrillar protein synthesis performed herein incorporates studies performed in healthy young individuals consuming a single, high-quality protein source (i.e., whey). While this approach increases homogeneity and allows for greater ease of comparison between studies, the results could be viewed as representing relative protein requirements under “ideal” conditions, notwithstanding the increased appreciation for the anabolic potential of whole foods (discussed in more detail below) (65–67). The following sections will briefly discuss conditions under which relative protein intakes may not be transferable and/or require further study.

### Exercise Modality

Dietary protein is important for the remodeling of skeletal muscle after not only resistance exercise but also after high-intensity sprint exercise (68), steady-state endurance exercise (69), and combinations thereof (i.e., concurrent training) (70, 71). Unlike resistance exercise, which provides a predominantly muscle-specific stimulus (72), endurance exercise can increase whole body oxidative disposal of amino acids that must ultimately be replaced via dietary sources (73). This may contribute to the increased protein requirements of endurance athletes (74, 75). Studies from the same laboratory utilizing identical tracer methodology have demonstrated that the ingestion of 0 g (~0.057 vs. ~0.051%/h, respectively), and 20 g (~0.087 vs. ~0.070%/h, respectively) of whey protein elicit broadly similar rates of myofibrillar protein synthesis after 90 min of endurance exercise (~77% maximal aerobic capacity), and traditional resistance exercise (23, 69), which could be interpreted as reflecting a similar post-exercise protein requirement after these dichotomous exercise stimuli. However, it has recently been demonstrated in a group design that post-exercise rates of myofibrillar protein synthesis were ~16% greater after the ingestion of 20 g (~0.27 g/kg) of milk protein (whey, casein, and milk protein concentrate) compared to a protein-free control after an acute bout of concurrent exercise (71), which is slightly lower than the reported ~32% difference in myofibrillar protein synthetic rates between 25 g of whey (~0.32 g/kg) protein and a protein-free placebo after concurrent exercise in a crossover study (70). Although the relative differences in myofibrillar protein synthetic rates between 0 g protein and a moderate relative intake (i.e., ~0.26–0.32 g/kg) in these concurrent exercise studies seem muted compared to the present post-resistance exercise analysis (i.e., ~16–32 vs. ~79%), the estimated increase from basal may be moderately more comparable (i.e., ~78–147 vs. ~152%; Figure 3). Therefore, while the consumption of ~ 0.31 g/kg of protein would enhance myofibrillar remodeling after all forms of exercise, additional research may be warranted to confirm that this represents a saturable dose and/or is sufficient to fully replace any endurance exercise-induced oxidative amino acid losses. This is notwithstanding the other potential benefits of increased protein ingestion in endurance athletes during periods of intensified training that may be dissociated from myofibrillar remodeling, such as enhanced immune function and/or exercise performance (76, 77).

### Population Age

Both young and old adults are capable of mounting an enhanced muscle protein synthetic response after resistance exercise in the fasted state (78, 79), which is consistent with the ability to increase muscle mass with this type of training across the lifespan (80). However, it has been observed that the combined effects of resistance exercise and amino acid ingestion on the enhancement of muscle protein synthesis may be delayed (81), and/or blunted in older adults (82, 83), suggesting nutrient sensitivities may be compromised with advancing age. In potential support, it has been shown that the ingestion of 40 g (~0.49 g/kg) of whey protein enhanced rates of post-exercise myofibrillar protein synthesis over and above that observed with 20 g (~0.25 g/kg) in older (~70 y) adults (82). However, the relative dose may not be substantially greater than younger adults as 30 g (~0.37 g/kg) of milk protein concentrate was recently demonstrated to enhance post-exercise myofibrillar protein synthetic rates in healthy older adults with no further benefit at 45 g (~0.56 g/kg) (84). Given that the anabolic potential of exercise and/or nutrition may be intimately tied to the “biological” age of a muscle as dictated by its habitual activity (85, 86), additional research is needed to confirm whether greater relative intakes are required to maximize post-exercise anabolism in older age and, if so, what lifestyle and/or biological factors may need to be considered (e.g., daily step count, presence/absence of sub-clinical chronic inflammation, excess body fat, etc.).

### Protein Type

The studies examining the post-exercise ingested protein dose-response utilized high quality (i.e., enriched in EAA), rapidly digested protein sources (i.e., egg and whey) (19, 23). Moreover, the estimates for the relative protein requirements derived herein were obtained with studies utilizing whey protein, which due to its rapid digestion (37), and/or greater leucine content (87, 88) elicits an early (i.e., within 3 h) and robust post-exercise stimulation of muscle protein synthesis. In contrast, proteins that contain lower quantities of the branched-chain amino acids (e.g., plant-based, caseinate), and/or are slowly digested (e.g., micellar casein) generally result in a suboptimal muscle protein synthetic response compared to an equal amount of whey protein (88), although recent research with dairy proteins may not support this “rapid rate of leucinemia” requirement for post-exercise myofibrillar remodeling (89). Nevertheless, studies have suggested that proteins with suboptimal essential amino acid and/or leucine content may ultimately be compensated for by ingesting a greater absolute protein amount. For example, it has been reported that the post-exercise stimulation of mixed muscle protein synthesis over 5 h (90), and myofibrillar protein synthesis over 3–5 h (34) of recovery is similar with the ingestion of ~20 g of a mixed protein (i.e., whey, casein, soy blend) and ~17 g of whey. Therefore, the optimal intake of proteins that may be relatively deficient in EAA and/or leucine and/or slowly digested may need to be addressed in future studies. Alternatively, individuals who prefer to ingest lower quality proteins (insofar as the stimulation of muscle protein synthesis is concerned) may consider consuming intakes at the upper “safe” intake of ~0.39 g/kg.

### Food Matrix

Early studies investigating the nutritional regulation of muscle protein synthesis have primarily provided dietary protein in beverage form. However, recent focus has been placed on the importance of studying whole foods (e.g., egg, beef) given these are typically nutrient-dense and arguably more representative of “normal” habitual dietary patterns (66, 67). Inasmuch as the peak and/or the rate of change in blood amino acid concentration regulates post-exercise muscle protein synthesis (37), the typically delayed digestion and absorption of solid foods may result in an attenuated muscle protein synthetic response (91). In this event, it is unclear if consuming a greater protein intake to account for any attenuated hyperaminoacidemia from solid food ingestion may be required to maximize post-exercise muscle protein synthesis. However, digestion rate may not be the only (or even primary) variable that influences the anabolic potential of whole food as minced beef has been demonstrated to induce a more rapid postprandial aminoacademia than skim milk but a lower early (i.e., <2 h), and potentially cumulative (i.e., 0–5 h) post-exercise myofibrillar protein synthetic response (92). Other studies have also demonstrated whole milk as more anabolic than skim milk (93) and skim milk more anabolic than soy juice (8) during post-exercise recovery. Finally, we recently demonstrated that whole egg supports a greater post-exercise myofibrillar protein synthetic response than an isonitrogenous quantity of egg white protein, which was supported by a greater lysosomal targeting of the mechanistic target of rapamycin (mTOR) as the potential underlying physiological mechanism (94, 95). This could suggest there may be circumstances whereby whole, nutrient-dense foods may require a lower relative intake to maximize post-exercise anabolism than other isolated protein sources. Although additional research is warranted to define the anabolic potential of whole food and its associated dose-response relationship to post-exercise anabolism, a target of ~0.31 g/kg protein could arguably represent a reasonable starting point for individuals aiming to enhance myofibrillar protein synthetic rates in the interim.

### Habitual Protein Intake

Although it is generally accepted that daily protein requirements are elevated in strength athletes (96), habitual intakes of populations engaged in chronic resistance training generally far exceed most recommendations (i.e., >2 g/kg/d) (97). Habitually high protein diets increase the capacity for protein catabolism and amino acid oxidation as a means to manage this excess macronutrient load (98). From an acute feeding standpoint, rodent models have demonstrated that adaptation to a high protein intake is accompanied by a greater splanchnic extraction of dietary nitrogen, which results in an attenuated post-prandial delivery to and deposition of dietary nitrogen in peripheral tissues (99). In this way, the gut may act as a buffer to ensure amino acid delivery to peripheral tissues (including muscle) is relatively constant regardless of habitual dietary protein intake. This has some support in humans as there is reduced dietary amino acid availability after consumption of 25 g of milk protein when adapted to a moderate (1.5 g/kg/d) as compared to low (0.7 g/kg/d) protein diet (100), suggesting a potentially greater splanchnic amino acid sequestration. Although rested postprandial rates of myofibrillar protein synthesis were non-statistically attenuated by ~50% in the moderate compared to the low protein group in the study by Gorissen et al. (100), Pasiakos et al. (101) demonstrated that the postprandial stimulation of mixed muscle protein synthesis by 20 g of protein was attenuated when consuming 1.6 vs. 0.8 g/kg/d and was not enhanced with a 2.4 g/kg/d controlled diet. Collectively these data could suggest that individuals habituated to lower protein diet approximating the recommended dietary allowance (RDA; 0.83 g/kg/d) may be able to support maximal rates of muscle protein synthesis after exercise with intakes lower than ~0.31 g/kg. In contrast, those adapted to higher habitual intakes, as is common in many strength athletes, may require a greater relative intake to account for an attenuated peripheral dietary amino acid appearance and/or enhanced amino acid oxidative capacity. However, the threshold at which this greater acute requirement may manifest could be relatively high (e.g., ~3x the RDA) given that previous post-exercise dose-response studies recruited participants with relatively high self-reported habitual intakes (i.e., 1.4–2.3 g/kg/d) yet still demonstrated approximate plateaus in muscle protein synthetic rates with 20 g (~0.24 g/kg) protein ingestion (19, 23).

### Negative Energy Balance

Muscle protein synthesis is an energetically expensive process and is down-regulated during periods of cellular energy stress, such as during a diet-induced negative energy balance (49, 102). The post-exercise stimulation of myofibrillar protein synthesis with dietary protein ingestion is not affected by low levels of muscle glycogen (103), highlighting that acute energy restriction does not constrain post-exercise muscle remodeling with exogenous amino acid ingestion. In contrast, more chronic periods of negative energy balance (i.e., 5–10 d) suppress resting mixed and myofibrillar protein synthesis (49, 102, 104). In addition, after a 5-day moderate protein (i.e., 1.4 g/kg/d) low energy (30 kcal/kg fat-free mass/d) diet, post-exercise myofibrillar protein synthesis is increased in a linear dose-dependent fashion with 15 and 30 g of dietary protein (49). Although the maximal absolute protein intake was lower than previous dose-response studies during energy balance (i.e., 30 vs. 40 g) (19, 23), there was no apparent plateau in post-exercise myofibrillar protein synthesis within the range of relative protein intakes studied (i.e., up to ~0.5 g/kg body weight) (49). Additionally, the estimated maximal myofibrillar protein synthesis with 30 g protein ingestion (determined by the group mean response) was ~82% above the rested fasted rate during energy deficit (49), which is less than the estimated plateau of ~142% during energy balance in the present review and could suggest a saturable protein intake was not provided during this negative energy balance. While it is possible that maximal rates of myofibrillar protein synthesis may generally be constrained during chronic diet-induced negative energy balance, the lack of a plateau and the relatively modest increase in myofibrillar protein synthesis with 30 g of protein could also suggest that the protein intake required to maximize post-exercise myofibrillar protein synthesis is slightly greater during a period of energy restriction. This would generally be in line with the observations that high daily dietary protein intakes (i.e., at least ~2 times the RDA) are required to maintain lean body mass and muscle protein synthesis during a negative energy diet with (104, 105) or without resistance exercise (101). Additional benefits for higher protein intakes during negative energy balance could be increased satiety and post-prandial thermogenesis (106), both of which would help support weight loss goals. Therefore, although it has been suggested that 0.25–0.3 g protein/kg body weight should be targeted after exercise in athletes aiming to maintain lean body mass during weight loss (107), the ~0.31 g protein/kg body mass determined herein could be viewed as a minimum intake with a safe intake closer to ~0.4 g/kg for individuals consuming a sub-optimal energy intake.

### Obesity

Beyond traditional derangements in glucose metabolism, it is becoming appreciated that excess body fat may also be an independent factor contributing to the dysregulation of muscle protein synthesis in obese populations (108). For example, obesity has been associated with a blunted myofibrillar protein synthetic response to dietary protein ingestion (i.e., 36 g or ~0.35 g/kg) (109), and resistance exercise (110). In addition, this anabolic resistance, which is not reported in relatively active obese individuals (i.e., ~7,400 steps/day) (111), may be exacerbated by inactivity (112), which suggests this anabolic resistance of obesity, similar to older adults (85, 86), has a strong lifestyle component to its manifestation and severity. Thus, inasmuch as this anabolic resistance extends to the post-exercise sensitivity to dietary amino acids, it could be argued that obese individuals may require a greater relative protein intake than their lean counterparts when normalized to the metabolically active lean body mass. However, studies used in the present analysis that yielded a relative protein intake of ~0.31 g/kg included participants of average body fat (~15%). Therefore, providing recommendations relative to total body mass would result in a greater dose per kg lean body mass in obese individuals (i.e., ~0.34 vs. ~0.41 g/kg lean body mass, respectively, assuming 30% body fat), which subsequently may be sufficient to overcome any obesity-related anabolic resistance.

## Practical Application of Acute Relative Protein Intakes

A single bout of resistance exercise can increase muscle protein synthesis for up to 24–48 h with the duration for which it is elevated influenced by training history of the athlete (13, 113) and the specific exercise stimulus (11), which ultimately factor into the general inability of single acute (i.e., <6 h) “snapshots” of myofibrillar protein synthesis to predict training-induced muscle hypertrophy (114). However, individuals who are able to support greater rates of myofibrillar protein synthesis over this 24–48 h post-exercise recovery period have been shown to experience greater training-induced gains in muscle hypertrophy (13). Given that individuals who engage in resistance training for the goal of enhancing muscle mass and/or muscle strength typically train 3–5 times per week (115), athletes are generally in some state of post-exercise recovery. Dietary protein consumed at any point during this prolonged 24–48 h recovery period would ultimately contribute to the remodeling of skeletal muscle. Outside of the response after a single meal, the pattern and distribution of dietary protein ingestion has been shown to influence muscle protein synthesis over 12–24 h both at rest (116) and after resistance exercise (28, 117, 118). For example, the repeated ingestion of 20 g of whey protein (~0.25 g/kg) at 3 h intervals has been shown to support the greatest rates of myofibrillar protein synthesis and whole body net protein balance over the 12 h after an acute bout of resistance exercise (28, 117). This has led to the suggesting that 4–5 meal occasions, which is the typical feeding frequency already adopted by many elite athletes (24), would be the most favorable and metabolically efficient means to consume one's daily protein intake if the goal is to maximize skeletal muscle remodeling while simultaneously minimizing irreversible amino acid oxidative catabolism (28, 117). Therefore, if one were to take a “muscle-centric” view for the daily protein requirement then the optimal amount and pattern of protein intake would translate into ~1.24–1.55 g/kg/d for a resistance-trained individual aiming to maximize skeletal muscle remodeling and/or net protein accretion. Even if one were to apply a conservative ~20% correction-factor (i.e., ~0.37 g/kg) to account for less anabolic proteins [e.g., plant-based; (88)], then this pattern of protein intake would provide ~1.48–1.85 g/kg/d. Both of these estimates are within the range of intakes suggested to maximize lean mass growth with training (119) and are in line with current sports science consensus recommendations for daily protein intake (96).

## Conclusion

The present review puts forth the argument that protein recommendations should be normalized to the body weight of an individual for a greater ease of translation of the dose that maximizes muscle protein synthesis and minimizes amino acid oxidation during the recovery from resistance exercise. Based on re-analysis of previously published literature, an intake of ~0.31 g/kg of high quality protein represents a suitable target to maximize myofibrillar protein synthesis during recovery from resistance exercise, regardless of sex, and quantity of active muscle mass. Though additional research is warranted to confirm whether acute protein requirements to maximize post-exercise rates of muscle protein synthesis are influenced by age, chronic energy status, and/or food matrix, a moderate intake of ~0.31 g/kg of high quality protein represents a good approximation for individuals of all body sizes aiming to efficiently enhance the repair, remodeling, and net synthesis of skeletal muscle tissue after resistance exercise.

## Author Contributions

DM wrote and approved the final version the manuscript.

### Conflict of Interest Statement

The author declares that the research was conducted in the absence of any commercial or financial relationships that could be construed as a potential conflict of interest.
